# Perceptions and Feedback of Medical Students regarding Simulation Based Learning at Dow Medical College Karachi

**DOI:** 10.12669/pjms.41.11.12045

**Published:** 2025-11

**Authors:** Tazeen Rasheed, Riya Bhagwan, Bader Faiyaz Zuberi, Shaheen Bhatty, Darshan Kumar, Faiza Sadaqat Ali

**Affiliations:** 1 Tazeen Rasheed Department of Medicine, Dow Medical College, Dow University of Health Sciences, Karachi, Pakistan; 2 Riya Bhagwan, Dow Medical College, Dow University of Health Sciences, Karachi, Pakistan; 3 Bader Faiyaz Zuberi OMI Hospital, Karachi, Pakistan; 4 Shaheen Bhatty Department of Medicine, Dow Medical College, Dow University of Health Sciences, Karachi, Pakistan; 5 Darshan Kumar Department of Medicine, Dow Medical College, Dow University of Health Sciences, Karachi, Pakistan; 6 Faiza Sadaqat Ali Department of Medicine, Dow Medical College, Dow University of Health Sciences, Karachi, Pakistan

**Keywords:** Simulation Based Learning (SBL), Medical students, Perception

## Abstract

**Objective:**

To determine the medical students’ perception and feedback of Simulation Based Teaching at Dow Medical College, Dow University of Health Sciences, Karachi.

**Methodology:**

This cross-sectional study was conducted in Dow Medical College, Dow University of Health Sciences, Karachi, Pakistan during the period December 2024 to January 2025. All final year students of DMC were given a chance to participate in this study. Online Google Form was created, and no personal data was collected. The questionnaire measured three domains of student’s perception to teaching at simulation lab.

**Results:**

A total of 112 students participated in the study. Satisfaction was defined if the mean score of a factor was ≥ 3.0. The mean satisfaction score with respect to the first domain which is related to SBL, simulation laboratory and instructors was 4.3 ±0.80. The mean score related to the second domain related to positive aspects of SBL was 4.29 ±0.70. Reverse coding was done for three questions in the third domain namely the negative aspects of SBL. The mean score of the third domain was found to be 2.61 ±1.01.

**Conclusion:**

Study reveal a favourable perception of medical students to SBL with regards to the teachers, the realism of the scenarios and the beneficial effects of SBL concerning its helpfulness in retaining medical knowledge, improving communication skills and confidence in dealing with future emergency situations. Our results also reveal a significant need in increasing the number of manikins’ and teachers/instructors at the simulation laboratory.

## INTRODUCTION

Simulation is taken from the Latin word ‘simulare’ meaning ‘to copy’.^1^ Clinical medical and surgical practice has benefited from new technologies such as realistic simulation (RS).^2^ A number of studies have highlighted the importance of inclusion of clinical research and simulation in medical curriculum in order to benefit students.^3^ RS requires recreating real life case scenarios which reflect real clinical practice, thereby improving students’ skills and decision-making powers within controlled environments. Simulation was first started in the ninth century by Madame du Coudray.^4^ Since 1960, Simulation based teaching have increasingly been used in high-risk professions like doctors, nurses, pilots, military personnel and lay-persons in resuscitation techniques.

In the past, medical education centred around observing doctors as they interacted with real patients during treatment and examinations. However, with the development of simulation-based teaching which is now being practised in different countries of the world, medical students get the chance to practice on manikins in a controlled environment. Simulations also help students to practice the application of knowledge in an environment that is devoid of any risks.^5^ This hands-on experience enhances the students’ communication and practical skills.^6^ Simulation based learning (SBL) is being practised in different specialities related to the field of medicine and surgery.^7^ Simulation provides students with a chance to learning practical experiences that connect theoretical knowledge with real-world application.^8^

Simulation-Based Medical Education (SBME) is a widely utilized training and assessment tool in undergraduate medical education.^9^ SBME instructional approaches frequently involve utilizing high fidelity manikins, and standardized patients to enhance learning experiences.^7^ As patients become more concerned that students and residents are gaining knowledge by practising on them, focus is now shifting on patient care and safety.^10^ Simulation-based assessments can also provide learners with immediate feedback, which can help them identify areas for improvement and guide their learning.^2^ Teachers can points out both the negative and positive practices and behaviours to the participants.

Understanding students’ perception with the new teaching method is crucial for incorporating simulation as a teaching and learning strategy in medical education curricula with the recent development of a new simulation lab at DMC, SBL has recently started in DMC in the subjects of Medicine, paediatric, gynaecology for third and final year students. The purpose of this lab is to provide a safe learning environment where students can perform on manikins observed by faculty and get feedback on their performance. Not much work is reported form Pakistan on this new modality of teaching, and there exists a gap in literature regarding simulation-based teaching especially in public sector medical colleges. The purpose of this study was to determine students’ perception and feedback with regards to SBL and to determine their views on its strengths and weaknesses and will thus help to improve the teaching and learning.

## METHODOLOGY

This cross-sectional observational study was conducted during the period December 2024 to January 2025. The study was conducted at Dow Medical College, Dow University of Health Sciences (DUHS). All final year students were offered the chance to participate.

### Ethical Approval:

It was taken from IRB of DUHS vide letter number: IRB-3702/DUHS/Approval /2024/348; Dated: 26-11-2024.

The items for the questionnaire were selected after a thorough literature review. Literature was searched using PubMed and Google scholar.^11^ Questionnaire was developed observing seven steps as per AMEE Guide #87.^12^ The seven steps are as below:

1. Conduct a literature review.
2. Conduct interviews and/or focus groups.
3. Synthesize the literature review and interviews/focus groups.
4. Develop items.
5. Conduct expert validation.
6. Conduct cognitive interviews.
7. Conduct pilot testing.

As the final seventh step was to conduct a pilot study on 30 students before it was distributed in its final form. The pilot study showed good internal and external validity and same questionnaire was used for study. The questionnaire consisted of a 5-point Likert scale showing degrees of agreement, with one being ‘strongly disagreed’ and 5 being ‘strongly agreed’. The questionnaire included the following three domains:

1. Questions related to SBL, simulation laboratory and instructors. 9 items (maximum score, 45)
2. Questions related to positive aspects of SBL: 8 items (maximum score, 40)
3. Questions related to negative aspects of SBL: 3 items (maximum score, 15)

### Inclusion & Exclusion Criteria:

All final year students at Dow Medical College (DMC) were offered to participate in this study, there were no exclusion criteria. Online Google Form was created based on the above-mentioned questionnaire and was forwarded by email using the college student database of mass email server after securing permission from Principal DMC. The students were required to fill in the forms anonymously, therefore separate consent forms were not required to be filled in by the students. Data was stored password protected on Google Drive automatically as the students responded, with only the first author having access to the data. The data was handled and stored in accordance with the tenets of the Declaration of Helsinki (1964, seventh edition in 2013). The submitted forms were then entered/imported in SPSS for analysis.

### Data analysis:

Descriptive statistics like gender as noted of all the students. The total scores and scores for individual domains were calculated. The mean ±SD of the total scores of the Likert items was calculated. The internal reliability of our survey was tested using Cronbach’s Alpha and a score of ≥ 0.7 was considered reliable. Means of scores were calculated and expressed with Standard Deviation (SD). Comparison of scores based on gender was done by Student’s t-test. Significance was set at ≤.05. Analysis was done using SPSS version 26.

## RESULTS

A total of 112 students participated in the study. Out of the 112 students 32 (28.6%) were males and 80 (71.4%) were females. The internal reliability of our survey was evaluated using Cronbach’s Alpha and was found as 0.921. A score of ≥0.7 was considered dependable. The questionnaire was divided into three domains (Table-I). The cut-off value for satisfaction was set at 3.0. Satisfaction was defined if the mean score of a factor was ≥3.0. The mean satisfaction score with respect to the first domain which is related to SBL, simulation laboratory and instructors was 4.3 ±0.80. The mean score related to the second domain related to positive aspects of SBL was 4.29 ±0.70. Reverse coding was done for three questions (Q18, 19, 20) in the third domain namely negative aspects of SBL (meaning 1=Strongly agree, 2= Agree, 3=Neither agree nor disagree, 4=Disagree, 5= Strongly disagree). The mean score of the third domain was 2.61 ±1.01.

**Table-I:** Mean score of 20 items in three domains from 112 students of DMC.

SN	QUESTIONS	SCORE ±SD
***Domain # 1: Questions related to SBL, simulation laboratory and instructors***		
1	Simulation is a useful addition to learning with real patients.	4.41 ±0.91
2	I am satisfied with the realism of the simulation scenarios.	4.11 ±1.17
3	The simulation environment is conductive to learning.	4.43 ±0.87
4	SBL should be included in the modules more frequently.	4.57 ±0.87
5	I was given the chance to practice adequately at the simulation laboratory.	4.09 ±1.21
6	I am satisfied with the level of expertise of the instructor at the simulation lab.	4.29 ±0.92
7	The time allocated for each simulation scenario is appropriate.	4.09 ±0.97
8	The facilities at the simulation laboratory are adequate.	4.43 ±0.97
9	The instructor gave proper instructions and feedback during the simulation scenario.	4.27 ±1.00
***Domain # 2: Questions related to positive aspects of SBL***		
10	SBL provides patient-like scenarios.	4.30 ±0.83
11	SBL helps me to get accustomed with the hospital environment.	3.96 ±1.19
12	Debriefing helps me to learn from my own mistakes.	4.46 ±0.73
13	SBL has made the subject more engaging.	4.54 ±0.68
14	SBL helps me to retain what I have learned in lectures.	4.50 ±0.74
15	SBL has improved my ability to interact more effectively with my colleagues.	4.29 ±0.84
16	SBL enhances my communication skills.	4.18 ±0.89
17	SBL helped me to deal with emergency scenarios in a proper way taking timely decisions.	4.07 ±1.07
***Domain # 3: Questions related to the negative aspects of SBL***		
18	The group of students is too big for proper learning.	3.13 ±1.34
19	There is a need for more manikins to match the number of students.	2.11 ±1.09
20	There is a need for more teachers/instructors at the simulation laboratory.	2.61 ±1.18

The total mean score was found to be 80.84 ±12.48. The mean score of all three domains were 4.29 ±0.80, 4.28 ±0.70 and 2.61 ±1.00, respectively. Comparison of responses on basis of gender was tested with Student’s t-test and was found that female students gave significant higher scores as compared to their males’ counterparts in domain #1, (*p* = .004) while in the other two domains the difference was not significant. Details are given in Table-II.

**Table-II:** Comparison of mean scores of domains with Student’s t-test.

	Gender	Mean ±SD	Sig.
Domain #1	Male	3.95 ±0.96	.004
	Female	4.44 ±0.69	
Domain #2	Male	4.11 ±0.97	.088
	Female	4.36 ±0.55	
Domain #3	Male	2.88 ±1.06	.082
	Female	2.51 ±0.97	
Total	Male	77.06 ±16.58	.043
	Female	82.33 ±10.15	

Significance ≤.05.

## DISCUSSION

The current study on the SBL environment highlights several strengths and areas for improvement. This comprehensive analysis provides invaluable insights into the recognition and value of SBL, resource allocation, and the collaborative academic culture within medical education. The current study highlights the importance placed on SBL by both students and faculty, reflecting a collective commitment to enhancing educational quality. This recognition aligns with findings from various published studies, which consistently highlight the benefits of SBL in medical education. The results of our study reveal a favourable perception of medical students to SBL with regards to the teachers, the realism of the scenarios and the beneficial effects of SBL concerning its helpfulness in retaining medical knowledge, improving communication skills and confidence in dealing with future emergency situations. This was evidenced by the mean score of 4.29 ± 0.80 and 4.28 ± 0.70 respectively in all questions related to the first two domains of our questionnaire.

The results also reveal a significant need in increasing the number of manikins’ and teachers/instructors at the simulation laboratory. Specifically, the data indicates a pressing requirement for more manikins to match the number of students, as evidenced by a mean score of 2.11±1.085. Additionally, there is a notable demand for more teachers and instructors, reflected by a mean score of 2.61 ±1.181. In a similar study done by Agha S, et al.^13^ 50% of the students reported that the use of simulation improved their clinical decision-making skills, retention of clinical knowledge and psychomotor skills.^11^ However, most of the students showed their dissatisfaction with the skills-laboratory facilities and the time allocated for skills-laboratory.

In a study by Saeed S et al, students scored higher if taught on SBL as compared to those conventionally.^14^ Similarly clinical performance of nurses was better when taught by SBL.^15^ The results of a study conducted by El Naggar MA, et al.^16^ were like ours, with regards to improvement in clinical skills, retention of knowledge. However, the challenges they reported were inadequate models, unsuitable laboratory rooms for training, inadequate skills laboratory facilities, and inappropriate time for laboratory activities. Coelho DL, et al.^17^ further support this by demonstrating that realistic simulations significantly improve medical students’ performance in advanced cardiac life support courses. However, they also emphasized the need for sufficient resources to facilitate these simulations. This is echoed by Sinha A et al,^18^ who argue that the quality and quantity of simulation tools directly impact the training and assessment in medical education.

In comparison Boonmak P et al.^8^ conducted a cross-sectional online national survey in Thailand and found that the primary challenges in simulation-based medical education include the time and space limitations, lack of equipment and trained personnel. This is consistent with our study’s results, which underscore the need for more manikins and teachers to enhance the simulation laboratory’s effectiveness.

Moreover, the ethical implications of simulation-based education, as discussed by Ziv A et al.^19^ point out that ensuring access to well-resourced simulation environments is not just beneficial but imperative for ethical medical training. The integration of simulated patients, as explored by Eklics K et al.^7^ also suggests that effective medical education requires carefully designed designs, diverse and realistic simulation experiences facilitated by adequate resources. Rajaure YS et al.^20^ conducted a comparative study to assess performance and confidence level of simulation based clinical examination in preclinical undergraduate medical students. They reported that students receiving both SBL and conventional teaching performed better than students receiving only conventional teaching alone.

They concluded that a structured curriculum should be designed to integrate simulation-based clinical examinations into preclinical medical students’ practical sessions.^17^ Over the past decades SBL has gained tremendous importance and is recognized as a valuable tool to expedite skill training and assessment with the shift in the paradigm in methods of teaching, stress in now being laid on protocol-based teaching and management of patients.^19^,^21^ For this patients having specific clinical conditions might be required. And such patients might not be always readily available. With SBL medical students can learn management of critical life-threatening situations in an organized manner and an environment that does not compromise the safety of the patients.^20^

In DUHS about 80% of enrolled students are female, this is also being reflected in results of our study which is heavily skewed in their direction. Thus, the opinion of male student is diminished in our study. This could be addressed by multicentre study with equal representation of both genders.

In summary, our study’s findings resonate with the published literature, underscoring a recurrent theme: the critical need for well-equipped simulation laboratories staffed with proficient instructors. Addressing these needs is paramount to optimizing the educational outcomes and ensuring that medical students are well-prepared for real-world clinical scenarios.

### Limitations

While this study provides valuable insights into the resource and staffing needs of the simulation laboratory, it is not without limitations. Firstly, the sample size was small, which might limit the generalizability of the findings to other institutions or settings. Secondly, the study relied on self-reported data from participants, which may be subject to response biases or inaccuracies. Finally, the study did not account for variations in the quality and usability of existing manikins and other simulation tools, which could influence the perceived need for additional resources. Future research should consider longitudinal approaches and larger, more diverse samples to build on these findings and address these limitations.

## CONCLUSION

The study reveals a favourable response of medical students to multiple aspects of SBL namely the teachers, the realism of the scenarios and the beneficial effects of SBL concerning its helpfulness in retaining medical knowledge, improving communication skills and confidence in dealing with future emergency situations. At the same time, it also highlights significant gaps in the resources and staffing of the simulation laboratory, with a pressing need for more manikins and instructors to effectively cater to the students’ needs. The mean scores obtained for the necessity of additional manikins and instructors, accompanied by their respective standard deviations, underline an urgent call for action.

These findings are in alignment with the broader literature, which consistently emphasizes the pivotal role of adequate resources and qualified personnel in simulation-based medical education. Addressing these deficiencies is crucial to ensuring that medical students receive high-quality training, preparing them for real-world clinical scenarios and upholding the ethical standards of medical education. Investments in simulation infrastructure and faculty development are paramount to optimize learning outcomes and enhance patient care.

### Declarations:

This study does not involve any personal, financial, or other conflicts of interest.

### Authors’ Contribution:

**TR, RB:** Conceived, designed, Literature search.

**BFZ:** Did data analysis, interpretations, critical review.

**SB:** Critical analysis**,** Gave final approval of the version to be published.

**DK, FSA:** Did critical revision. Analysis

All authors are responsible for the integrity of the study.
